# Supplementary material for: Comparison of the results of in-person and mobile phone surveys for a health facility assessment in Tajikistan: A validation study protocol
Source: PLoS One. 2025 May 29;20(5):e0309570. doi: 10.1371/journal.pone.0309570 (PMC12121742; doi:10.1371/journal.pone.0309570)
Supplement: S1 Appendix — (DOCX) [file pone.0309570.s001.docx]

**SUPPORTING INFORMATION**

Appendix One. Indicator mapping

| **Survey Module** | **Indicator mapping type** | **Question**  **Rapid phone HFA (FASTR)** | **Question  Comprehensive in-person HFA with verification (SDI)** |
| --- | --- | --- | --- |
| SERV | [Question] | Does the facility offer the following services? | In [**last 3 months]**, was this health facility able to provide the following services? |
| SERV | Indirect | Integrated Management of Child Illnesses (IMCI) | Diagnosis and treatment of sick child/IMNCI |
| SERV | Indirect | Child nutrition services | Prevention, diagnosis and rehabilitation in child nutrition |
| SERV | Indirect | Consultations on healthy lifestyle and nutrition | Consultations on healthy lifestyle and nutrition |
| SERV | Indirect | Family planning services | Family planning services |
| SERV | Indirect | Antenatal care (ANC) services | Antenatal care (ANC) services |
| SERV | Indirect | Immunization of children | Childhood immunization |
| SERV | Indirect | HIV/AIDS | -HIV/AIDS counseling and testing  -HIV/AIDS care and support services |
| SERV | Indirect | Tuberculosis | -Tuberculosis diagnosis  -Tuberculosis treatment |
| SERV | Indirect | Hypertension | -Hypertension diagnosis  -Hypertension treatment services |
| SERV | Indirect | Diabetes | -Diabetes diagnosis -Diabetes treatment |
| SERV | Indirect | Chronic respiratory diseases (asthma, COPD) | Chronic respiratory diseases (asthma, COPD) diagnosis  Chronic respiratory diseases (asthma, COPD) treatment |
| SERV | Indirect | Child and Adult Survivors of Sexual Violence Care and Clinical Treatment Services (Domestic and Sexual Violence Victim Assistance Training) | In the last three months, was this health facility (RHC) able to provide any clinical treatment services for the following groups of victims/ survivors of gender-based violence?  i. Child  ii.Adolscents  iii.Adults |
| SERV | Direct | Does your facility open at different days and / or times for summer time versus winter time? | Does your facility open at different days and / or times for summer time versus winter time? |
| SERV | Indirect | On average, how many days a week is this facility open for outpatient services? | What days of the week is this health facility open? |
| SERV | Indirect | On the days the facility provides services, how many hours per day is this facility open? | -What time does this health facility open and close for OPD care from Mondays to Saturdays?  - What time does this health facility open and close for OPD care on Sundays? |
| SERV | Indirect | On the days the facility provides the service, how many hours per day is this facility open in the following times?  i.Summer time | - What time does this health facility open and close for OPD care in Summer Time from Mondays to Saturdays?  - What time does this health facility open and close for OPD care in Summer Time from Mondays to Sundays? |
| SERV | Indirect | On the days the facility provides the service, how many hours per day is this facility open in the following times?  ii.Winter time | - What time does this health facility open and close for OPD care in Winter Time from Mondays to Saturdays?  - What time does this health facility open and close for OPD care in Winter Time from Mondays to Sundays? |
| INF | Direct | What is the main water supply for this facility? | What is the main water supply for the health facility? |
| INF | Indirect | Is the main source of water supply located on the facility premises (in the building, or within facility grounds)? | Where is the main water supply for the health facility located? |
| INF | Direct | In the last 3 months, was there any time when the facility did not have water available? | In the last 3 months, was there any time when the health facility did not have water available for use? |
| INF | Direct | What type of toilet(s) are within the health facility premises for use by staff and patients? | Please state the type of toilet(s) that are within health facility premises for use by staff and patients |
| INF | Direct | Is there at least one improved, functional, accessible, and private toilet designated for Health Facility staff only? | Is there at least one improved, functional, accessible, and private toilet designated for health facility staff only? |
| INF | Direct | Is there at least one improved, functional, accessible, and private toilet designated for Females only? | Is there at least one improved, functional, accessible, and private toilet designated for females only? |
| INF | Indirect | Is there a bin with a lid for disposal of used menstrual hygiene products available in the toilet for females only? | Is there a bin with a lid for disposal of used menstrual hygiene products available in at least one improved, functional, accessible, and private toilet for females only? |
| INF | Indirect | Is there a hand-washing facility with running water and soap within 5m of the toilets? | Does this health facility have a functional sink with tap, water tank with tap, bucket with tap or other similar device enabling access to running water and soap together within 5 meters of the toilets? |
| INF | Indirect | Is there a hand-washing facility with either running water and soap or an alcohol-based hand sanitizer, available at points of care in this facility?  INTERVIEWER: Points of care are locations where diagnostic or therapeutic services, examinations, or consultations are provided to patients, such as patient consultation rooms. | Points of care are locations in the health facility where diagnostic or therapeutic services, examinations, or consultations are provided to patients, such as patient consultation rooms. A functional hand hygiene facility is any device that enables staff and patients to clean their hands effectively using running water and soap, or alcohol-based hand rub (such as a fixed or portable dispenser). Is there a functional hand hygiene facility at points of care in this **health facility**? |
| INF | Direct | Does this facility have a cleaning record that keeps track of when and by whom spaces, surfaces, and equipment are cleaned and disinfected? | Does this facility have a cleaning record that keeps track of when and by whom spaces, surfaces, and equipment are cleaned and disinfected? |
| INF | Indirect | What method does this health facility use for the final disposal of infectious medical waste other than sharps waste (e.g., used bandages and placentas)? | What method does this health facility use for the final disposal of infectious medical waste other than sharps waste (e.g., used bandages and placentas)? |
| INF | Direct | What is the health facility’s main source of electricity? | What is the health facility’s main source of electricity? |
| INF | Direct | In the last 3 months, was there any time when this facility did not have electricity while the facility was open for services? | In the last 3 months, was there any time when this health facility did not have electricity supply while the facility was open for services? |
| INF | Indirect | Does this facility have the following communication systems, available in functioning condition today:   1. Functional telephone | - Does this health facility have at least one landline telephone that is available to call outside at all times?  - Is at least one landline telephone functional today?  - Does this health facility have at least one regular mobile/cellular telephone (not a smartphone) or does a health facility staff member have a private cellular telephone (not smartphone) that is paid for by the health facility or district?  - Is at least one regular mobile/cellular telephone (not a smartphone) functional today?  - Does this health facility have at least one smartphone or does a health facility staff member have a private smartphone that is paid for by the health facility or district?  - Is at least one smartphone functional today?  - Does at least one regular mobile/cellular telephone or a smartphone have network coverage today? |
| INF | Indirect | Does this facility have the following communication systems, available in functioning condition today:   1. Functional computer | - Does this health facility have at least one computer?  - Is at least one computer functional today? |
| INF | Indirect | Does this facility have the following communication systems, available in functioning condition today:  c. General access to internet | - Does this health facility have at least one internet connection?  - Is at least one internet connection functional today? |
| INF | Direct | In the last 3 months, was there any time when the facility did not have any telephone service whether landline or mobile? | In the last 3 months, was there ever a time when this health facility did not have any telephone service (landline/mobile/cellular)? |
| INF | Indirect | In the last 3 months, was there any time when the facility did not have internet available (internet outages/disruptions)? | In the last 3 months, how many internet connection interruptions did this health facility experience? |
| INF | Indirect | Does this facility have access to at least one functional ambulance (able to start and move) or other four-wheeled motor vehicle provided by the health facility for emergency transportation, that is stationed at this facility or that the facility can call for? | Does this health facility have at least one ambulance or other four-wheeled motor vehicle for emergency transportation stationed at this health facility or that the facility can call for? |
| INF | Indirect | Is there currently an emergency vehicle available, in working order, and with fuel and a driver? | -Is at least one ambulance or other four-wheeled motor vehicle provided by the health facility for emergency transportation functional (able to start and move) today?  -Is there fuel available today for at least one ambulance or other four-wheeled motor vehicle provided by the health facility for emergency transportation?  -Is there at least one ambulance driver on duty today? |
| INF | Direct | National notifiable diseases refer to diseases of public health priority for monitoring and control efforts by the Ministry of Health.  Does this facility report cases of any national notifiable diseases to higher-level authorities? | Does this health facility report cases of any national notifiable diseases to higher-level authority/ies? |
| INF | Direct | Does this facility have infection prevention and control (IPC) guidelines? | Does this facility have infection prevention and control (IPC) guidelines? |
| INF | Direct | In the last 2 years, have any health care providers in this facility received in-service training on IPC? | In the last 2 years, has at least one health care provider in this facility received in-service training on IPC? |
| HR | Indirect | How many Family medicine doctors are working at this health facility? | Based on the health facility’s staff roster(s), what is the total number of the following health care providers working at this health facility (RHC/HH)?  i.Family Medicine Doctors |
| HR | Indirect | How many other doctors are working at this health facility? | Based on the health facility’s staff roster(s), what is the total number of the following health care providers working at this health facility (RHC/HH)?  ii.Other Doctors |
| HR | Indirect | How many Family medicine nurses are working at this health facility? | Based on the health facility’s staff roster(s), what is the total number of the following health care providers working at this health facility (RHC/HH)?  iii.Family Medicine Nurses |
| HR | Indirect | How many Midwives are working at this health facility? | Based on the health facility’s staff roster(s), what is the total number of the following health care providers working at this health facility (RHC/HH)?  iv.Midwives |
| HR | Indirect | How many other nurses (including Medical and Social Patronage nurses and treatment room, immunoprophylaxis, dressing room nurses) are working at this health facility? | Based on the health facility’s staff roster(s), what is the total number of the following health care providers working at this health facility (RHC/HH)?  v.Other Nurses (including Medical and Social Patronage nurses and treatment room, immunoprophylaxis, dressing room nurses) |
| HR | Indirect | How many pharmacists are working at this health facility? | Based on the health facility’s staff roster(s), what is the total number of pharmacists working at this health facility? |
| HR | Indirect | How many other clinical staff are working at this health facility? | Based on the health facility’s staff roster(s), what is the total number of Other clinical staff working at this health facility? |
| HR | Indirect | How many junior medical staff are working at this health facility? | Based on the health facility’s staff roster(s), what is the total number of Junior medical staff working at this health facility? |
| HR | Indirect | How many non-medical support staff are working at this health facility? | Based on the health facility’s staff roster(s), what is the total number of non-medical support staff working at this health facility? |
| HR | Direct | Does this facility have any protocols to increase the number of staff if needed? For example, calling in off-duty, or retired staff, temporarily employing volunteers or students, or repurposing non-clinical staff? | Does this health facility have any of the following guidelines/protocols in place in the case of a disaster/public health emergency?  Protocols to increase number of staff, such as calling in off-duty, retired, or volunteer staff and repurposing non-clinical staff as appropriate |
| SUP | [Question] | Are the following pieces of equipment currently available and functional? | Please tell me if the following are available in this health facility today. |
| SUP | Direct | Hemoglobinometer | Hemometer |
| SUP | Direct | Blood glucose meter | Blood glucose meter |
| SUP | Direct | Centrifuge | Centrifuge |
| SUP | Indirect | Refrigerator with thermostat | Is at least one refrigerator for the storage of vaccines functional today? |
| SUP | Direct | Child weighting scale | Weighing scale for children |
| SUP | Direct | Thermometer | Medical Thermometer |
| SUP | Direct | Blood pressure apparatus |  |
| SUP | Direct | Otoscope | Otoscope |
| SUP | Direct | Obstetric stethoscope | Obstetric stethoscope |
| SUP | Direct | Pulse oximeter | Pulse oximeter |
| SUP | Direct | Microscope | Microscope |
| SUP | Indirect | Oxygen delivery device | Oxygenator |
| SUP | [Question] | Are the following diagnostics or supplies available for any patient who needs it, available in a limited supply, or currently not available on-site? | In the last three months, was this health facility able to provide the following services? |
| SUP | Indirect | Dipsticks for urine protein, glucose, and ketone bodies | General urine dipstick test including urine protein, glucose, and ketone bodies |
| SUP | Indirect | Dipstick for urine protein | Dipstick test for urine protein |
| SUP | Indirect | Dipstick for glucose | Dipstick test for glucose |
| SUP | Indirect | Dipstick for ketone bodies | Dipstick test for ketone bodies |
| SUP | Indirect | Onsite blood glucose testing | -Urine test for glucose  -Blood glucose level using a glucometer  -Blood glucose (Biochemistry) |
| SUP | Indirect | Onsite malaria diagnostic testing | -Malaria Rapid Diagnostic Test  -Examination of malarial blood in a thick smear |
| SUP | Indirect | Onsite HIV diagnostic testing | -HIV (rapid diagnostic test)  -HIV serology |
| SUP | Indirect | Urine pregnancy test kit | Urine pregnancy test |
| SUP | Indirect | Onsite Syphilis testing | -Syphilis – Rapid Diagnostic Test (RDT) or specific assay kit  -Syphilis serology - pale treponema hemagglutination (TPHA) |
| SUP | Indirect | Onsite Blood hemoglobin testing | Blood haemoglobin testing |
| SUP | Indirect | Onsite Hepatitis B diagnostic testing | -Hepatitis B Rapid Diagnostic Test (RDT)  -Serology for hepatitis B (HBV) |
| SUP | Indirect | Onsite Hepatitis C diagnostic testing | -Hepatitis C Rapid Diagnostic Test (RDT)  -Serology test for hepatitis C (HCV) |
| SUP | [Question] | Are the following PPE available for all consultations that require them, available in a limited supply, or currently not available on-site? | Please tell me if the following are available in this health facility today. |
| SUP | Direct | N95 mask | N95 maks |
| SUP | Direct | Surgical or medical masks | Surgical or medical masks |
| SUP | Direct | Disposable latex gloves | Disposable latex gloves |
| SUP | [Question] | Are the following essential medicines currently available on-site? | Please tell me if the following are available in this health facility today. |
| SUP | Direct | Glucose solution | Glucose solution 5% 400 ml |
| SUP | Direct | Ringer's solution | Ringer's solution 400 ml |
| SUP | Direct | Sodium chloride solution | Sodium Chloride solution 0.9% 400 ml |
| SUP | Direct | Analgesics (e.g. Metamizole or Paracetamol) | -Metamizole sodium (Analgin) 50% 2 ml  -Paracetamol |
| SUP | Direct | Dexamethasone | Dexamethasone 4 mg 2 ml |
| SUP | Direct | Hydralazine | Hydralazine |
| SUP | Direct | Oxytocin | Oxytocin |
| SUP | Direct | Nifedipine | Nifedipine 10 mg |
| SUP | Direct | Drotoverin (No-shpa) | Drotoverine (No-spa) 40 mg |
| SUP | Direct | Magnesia sulfate | Magnesia sulfate 25% 10 ml |
| SUP | Direct | Adrenaline hydrotartrate | Adrenaline hydrotartrate 0.1% 2 ml |
| SUP | Direct | Nitroglycerin | Nitroglycerin 5 mg |
| SUP | Direct | Mezaton | Mezaton 1% 1 ml |
| SUP | Direct | Ammonia | Ammonia 10% 25 ml |
| SUP | [Question] | Are the following vaccines available for any patient who needs it, available for only some patients, or currently not available on-site? | Please tell me if the following vaccines, injections, and IV fluds are available in this health facility today |
| SUP | Direct | Measles, mumps and rubella vaccine (MMR) | MMR (measles-mumps-rubella vaccine) |
| SUP | Direct | Penta (DPT + HiB + HepB) vaccine | Pentavalent vaccine (DTP, H ib , hepatitis B) |
| SUP | Direct | BCG vaccine | BCG vaccine |
| SUP | Direct | Polio vaccine | Oral polio vaccine (OPV) |
| SUP | Direct | PCV (pneumococcal vaccine) | PCV (pneumococcal vaccine) |
| LC | Direct | In the past three months, for outpatient referrals **from** this facility for care at a higher-level health facility, what were the main reason for referral? | In the last 3 months, for outpatient referrals this health facility for care at a **higher-level** health facility, what were the **3 most common** reasons for referral? |
| LC | Direct | In the past three months, for outpatient referrals **to** this facility from a lower-level health facility, what were the main reason for referral? | In the last 3 months, for outpatient referrals to this health facility from a lower-level health facility, what were the 3 most common reasons for referral? |
| LC | Indirect | A supervision visit occurs when external supervisors from higher regulatory or supervisory bodies visit a health facility to assess the quality of clinical work, ensure compliance with fire safety regulations, control cleanliness, etc. This does not include clinical visits made by family medicine doctors.  In the last 12 months, were any visits for the purpose of supervision carried out in this facility? | A supervision visit occurs when external supervisors from higher regulatory or supervisory bodies visit a health facility to assess the quality of clinical work, ensure compliance with fire safety regulations, control cleanliness, etc. This does not include clinical visits made by family medicine doctors.  In the last 12 months, how many supervision visits did this health facility receive? |
| LC | Direct | In the last 12 months, who conducted the last supervision visit? | In the last 12 months, who conducted the last supervision visit? |
| LC | Direct | Supportive supervision promotes open, two-way communication, teamwork for problem solving, and goal monitoring. Supportive supervision is conducted to improve/support clinical care, compliance with guidelines, quality improvement, and so on.  Would you consider the last supervision visit to be "supportive"? | Supportive supervision promotes open, two-way communication, teamwork for problem solving, and goal monitoring. Supportive supervision is conducted to improve/support clinical care, compliance with guidelines, quality improvement, and so on.  Would you consider the last supervision visit to be "supportive"? |
| LC | Direct | Does this facility have a focal person or team that has clearly defined roles and responsibilities in the event of a disaster or public health emergency? | Does this health facility have a focal person or team that has clearly defined roles and responsibilities in the event of a disaster or public health emergency? |
| LC | Direct | Does this facility currently have any of the following guidelines or protocols for communication during a disaster or public health emergency?  a. Protocols for emergency communication between this facility and higher-level authorities | Does this health facility have any of the following guidelines/protocols for communication during a disaster/public health emergency?  a. Protocols for emergency communication between this facility and higher-level authorities |
| LC | Direct | Does this facility currently have any of the following guidelines or protocols for communication during a disaster or public health emergency?  b. Protocols for emergency communication between this facility and other health facilities | Does this health facility have any of the following guidelines/protocols for communication during a disaster/public health emergency?  b. Protocols for emergency communication between this facility and other health facilities |
| LCD | Direct | Does this facility currently have any of the following guidelines or protocols for communication during a disaster or public health emergency?  c.Protocols for emergency communication between this facility and its catchment population or the public | Does this health facility have any of the following guidelines/protocols for communication during a disaster/public health emergency?  c.Protocols for emergency communication between this facility and its catchment population or the public |
| COM | Direct | A Community Health Team refers to a group of individuals representing diverse interests within the community, who work together to achieve a common goal of improving health service delivery and health outcomes. Does this facility have a community health team? | A Community Health Team refers to a group of individuals representing diverse interests within the community, who work together to achieve a common goal of improving health service delivery and health outcomes. Does this facility have a community health team? |
| COM | Direct | In the last 12 months, how often did this facility's Community Health Team meet? | In the last 12 months, how often did this facility's Community Health Team meet? |
| QI | Direct | Quality improvement refers to changing how health services are delivered to make them more effective, safe, and/or people-centred.  In the last 12 months, did this facility conduct **any** quality improvement activities for any service areas? | Quality improvement refers to changing how health services are delivered to make them more effective, safe, and/or people-centred.  In the last 12 months, did this facility conduct **any** quality improvement activities for any service areas? |
| QI | Indirect | Does this facility have a focal person, committee, or team for quality improvement and patient safety? | In last 12 months, who was responsible for conducting quality improvement activities in this health facility? |
