# Supplementary material for: Comparison of the results of in-person and mobile phone surveys for a health facility assessment in Tajikistan: A validation study protocol
Source: PLoS One. 2025 May 29;20(5):e0309570. doi: 10.1371/journal.pone.0309570 (PMC12121742; doi:10.1371/journal.pone.0309570)
Supplement: Attachment — Submitted filename: Response to the reviewers - PONE-D-24-33961.docx [file pone.0309570.s004.docx]

Daisuke Nagasato

Academic Editor

PLOS ONE

6 March, 2025

Re: Response to the reviewers for PONE-D-24-33961: Comparison of the results of in-person and mobile phone surveys for a health facility assessment in Tajikistan: a validation study protocol

Dear Dr. Nagasato,

Thank you for your time and review of our paper, “Comparison of the results of in-person and mobile phone surveys for a health facility assessment in Tajikistan: a validation study protocol”. We have responded to each of the reviewer’s questions below and in the attached track change version of the manuscript. Please note that the page numbers below refer to the page numbers in the track changed copy of the manuscript.

In addition, we have aligned the funding statements from the manuscript and the online submission portal. The two surveys are being funded by the World Bank and the Global Financing Facility for Women, Children, and Adolescents; however, there is not specific grant number as these studies are a part of our routine support to the Government of Tajikistan. We have done our best to reflect this accurately in the portal.

Further, we have added the IRB information from the portal into the manuscript itself, to align with the requirements of the Inclusivity in Global Health Research Questionnaire.

Finally, to reflect the work on the revisions, we have changed the author order on the submission. Pablo Fernandez and Rachel Neill are still listed as co-first authors, but we have switched the order that the names appear in the manuscript.

We look forward to hearing from you on our submission.

Best regards,

Rachel Neill

Corresponding author

**Reviewer's Responses**

**Reviewer #1:**

The authors describe the protocol for a study to assess the reliability and validity of a rapid phone-based Health Facility Assessment (HFA) in Tajikistan via a comparison against a comprehensive in-person HFA (here used as the reference standard). This is of interest because phone surveys entail cost-savings and could be done more frequently, producing more timely data.

The literature review is comprehensive and the protocol is well described. Below I have listed a few (minor) comments that may improve the manuscript:

*Thank you for your time and review of our manuscript. We appreciate your feedback below and have incorporated each of your comments into the manuscript.*

(1) Describe the plans for data storage and the modalities for (future) public access of the data.

*Our intention is to make the data from both surveys publicly available on the survey’s respective microdata catalog/ repository, with approval from the Government of Tajikistan and in alignment with the World Bank’s data privacy guidelines which ensure personally identifiable information is not released. Datasets would be posted on (1) the Service Delivery Indicators Microdata Library (https://microdata.worldbank.org/index.php/catalog/sdi/?page=1&ps=15&repo=sdi ), and (2) the FASTR Resource Repository (*[*https://data.gffportal.org/key-theme/FASTR/resource-repository/index.php/home*](https://data.gffportal.org/key-theme/FASTR/resource-repository/index.php/home)*). Data stored here is hosted on World Bank servers and is available for public access indefinitely. We have made note of this in the manuscript on page 23, as well as the PlosOne submission portal.*

(2) P6: the evidence linking interview duration to data quality is mixed, e.g., <https://doi.org/10.1177/1525822X241237042>

*Thank you for sharing this article, which is very useful for our work. We have added this into the introduction, please see pg.6 in the track change version of the manuscript. Please note that the citation has only been updated in the clean version of the manuscript due to issues with syncing our citation library (it is citation 18 in the clean version).*

(3) I wonder whether informed consent in required given that this is not human subjects research in the strict sense. Respondents report on a public service and not on their own attributes or behavior.

*This is a great question. We submitted, and received IRB approval for, taking informed consent from all participants despite the fact that our research is not in the strict sense human subject research. We have therefore left this unchanged in the manuscript.*

(4) P20 what are ‘heterogeneity effects due to facility characteristics’ ?

*We were referring to any differences in the surveys that can be attributed to facility characteristics. We have modified the manuscript by removing that term and replacing it to read, “Regression analysis will also be conducted to identify any variations in the findings that can be attributed to facility characteristics.”* *Please find this edit on pg. 21.*

(5) P20, L408: correct typo the “predefined” validation threshold …

*Thank you for catching this! We have fixed it in the manuscript. The edit is on the top of pg. 21.*

**Reviewer #2:**

The authors have written. A clear protocol to understand the potential validity of phone facility assessments versus traditional in person assessments. This is an important area of work as understanding and improving care delivery must include more rapid and census level facility assessments which is not feasible given the cost and time of in person facility assessments.

*Thank you for your time and review of our manuscript. We really appreciate your feedback which has made the protocol stronger and have incorporated each of your comments into the manuscript.*

The methods need a bit more clarification and overall, the term “phone-based HFA against a comprehensive in-person HFA.” Is a bit misleading as this is not comparing phone based versus comprehensive but phone be versus a subset of questions in one of 3 components of the comprehensive HFA. For example, understand quality (in SDI via vignettes) could be done by phone, and discussion how the 1 component was chosen is needed and adding as a limitation.

*On the comprehensive component: this is an important distinction, and we agree completely with your suggestion. We have modified the manuscript in the following places:*

- *Clarified the scope of the study on only the health facility questionnaire on pg. 10*
- *Emphasized this again with a rational on pg. 11*
- *Clarified that the comprehensive HFA was restricted in scope compared to the full SDI survey platform, but still more comprehensive than the phone survey as it contains 129 survey items compared to the phone-based HFA’s 102 survey items on the overlapping domains (pg. 13)*

It would have also been helpful if there was any exploration whether the shorter number of questions give similar results o strength of the readiness in different areas (supply, HR etc) which would help in monitoring change after interventions to address gaps. Some more details below
*This is a great point and actually something we were planning to explore as well. We have decided not to add this to the protocol to maintain brevity and promote clarity, but we will take this suggestion on board as a secondary analysis when we have the data. Thank you!*

Introduction’
While it is true that these rapid and more targeted assessment are important in fragile and conflict settings, they are critical to the broader effort in strengthening health systems and the quality of care delivered.

*True – and also reflective of our work and how we are deploying these surveys. We have edited this on page 5 in the track change version.*

They note the 2 HF phone surveys but need to explain what 70% sensitivity for the study in Malawi means.

*Thank you for catching that. we have modified to say, “The study found that most health facility indicators collected by phone were above their predetermined 70% sensitivity threshold to assess validity compared to in-person records and inspections and determined that phone data collection was both feasible and highly cost-effective”.* *See pg. 5*

The remainder of the challenges with phone surveys is well detailed (although is there evidence that lower nutritional intake was due to fatigue or potential if there was understanding of the impact of the survey (more food). It is also not clear why a phone survey needs to be only with one person versus more than one call for a facility or having it as a group call

*This is a fair suggestion, and we have modified the text to clarify that this is a working hypothesis/ reflective of our practical experience implementing phone surveys. We have not been able to identify published evidence on these questions, demonstrating the value of more research on phone-based health facility survey approaches. This has now been clarified on page 7.*

When they look for objective 3, it would also be important to look at phone coverage which in some areas mya also be a barrier as well as cost to the recipient

*Great suggestion, we have added this on pg.* 21-22.

Methods
The rapid survey should be included in an appendix. A table also mapping where the survey measures versus the HFA.

*Absolutely. We have added the rapid phone survey tool as a supplementary attachment/appendix . The full mapping of survey questions across both questions is in Appendix.*

The plan to randomize order is well designed

*Thank you*

It would be helpful to understand why some questions were altered (like able to provide versus provide HIV services)

*This is a great question. In the end, we decided to maintain as much fidelity to our original instruments (the FASTR and SDI surveys) as possible in the study. For example, for the phone survey, we focus on questions that are as straightforward as possible without the need for enumerator instructions or explanation which does not always result in a perfect comparison to the comprehensive HFA questions. This is a limitation of the study, so we have added a strengths and limitations section on pg. 23 and explained this further.*

If the OIC decides they are not able to answer a question and delegate it to someone else-is this possible in the phone survey?

*In theory yes this is possible. Our data collection protocol specifies that the OIC is the respondent, but if the OIC asks a question of others or delegates a response, we will accept that during the data collection.*

Curious why there is advance sharing of a questionnaire for the in person but not the phone survey?
*The main reason for the sharing of parts of the CAPI SDI questionnaire as a PAPI in advance of the in-person interview is because there is a verification process which occurs during that interview. We ask to see the actual documentation for a variety of items (for example, patient registration records and written examples of reports from supervisory visits) and if they do not know that they are needed, it can take significant time to pull / locate them. That visual verification is not possible during the call, so there was no need to share the questionnaire in advance.*

In the analysis-there is no specific plan to analyze differences in responses based on order of administration which is an important methodology plan.

*Agree – this is also in our plan and was inadvertently not included in the manuscript. This has been added on pg. 21.*
